# Ovarian sensitivity index affects clinical pregnancy and live birth rates in gonadotropin-releasing hormone agonist and antagonist *in vitro* fertilization cycles

**DOI:** 10.3389/fendo.2024.1457435

**Published:** 2024-12-13

**Authors:** Chao Chin Hsu, Isabel Hsu, Sonam Dorjee, Yi Chun Chen, Tzu Ning Chen, Yu Lin Chuang

**Affiliations:** ^1^ Taiwan United Birth-Promoting Experts Fertility Clinic, Tainan, Taiwan; ^2^ Department of Obstetrics and Gynecology, National Taiwan University Hospital, Taipei, Taiwan

**Keywords:** GnRH-agonist, GnRH-antagonist, ovarian sensitivity index, controlled ovarian hyperstimulation, *in vitro* fertilization, clinical pregnancy rate, live birth rate

## Abstract

**Objectives:**

This study aimed to investigate the correlation of ovarian sensitivity index (OSI) and clinical parameters in IVF treatments.

**Methods:**

IVF data files between January 2011 and December 2020 in a single unit were included. The primary outcome measure was the correlation between the OSI and clinical pregnancy and live birth rates. A generalized linear model was employed to assess group differences while controlling for age. Correlations between the OSI and clinical parameters were analyzed using Pearson’s correlation test.

**Results:**

In total, 1,627 patient data were reviewed, comprising 1,160 patients who received GnRH antagonists and 467 who received GnRH agonists. There was no difference in the incidence of premature ovulation and LH surge in women receiving either GnRH antagonists or agonists. A higher number of mature oocytes and good embryos were obtained in the GnRH agonist cycles. No differences were observed in pregnancy and live birth rates between both groups. Regarding the correlation of the OSI with clinical parameters, serum anti-Müllerian hormone, cycle day 2 follicle-stimulating hormone, LH, and estradiol concentrations, numbers of larger follicles, fertilization rate, and the incidence of premature LH surge were positively correlated with the OSI. Whereas the body mass index, mature oocytes obtained, embryo transfer number, and dose of GnRH antagonists were negatively correlated with the OSI. In the GnRH antagonists group, an OSI of 225.75 significantly distinguished pregnancy from non-pregnancy (p < 0.001), with an AUC of 0.615, and an OSI of 208.62 significantly distinguished live births from non-live births (p < 0.001), with an AUC of 0.637. As for the GnRH agonist group, an OSI of 228 significantly distinguished live births from non-live births, (p =0.020) with an AUC of 0.569.

**Conclusion:**

We demonstrated the capability of employing OSI to distinguish the clinical pregnancy and live birth outcomes in IVF cycles.

## 1 Introduction

The efficiency of controlled ovarian hyperstimulation (COH), which directly affects the outcomes of treatments, including clinical pregnancy and live birth rates, is a major objective for assisted reproduction. Currently, personalized treatment based on an individual’s response to exogenous gonadotropin (Gn) is the main focus in clinical practice. The goal of COH is not only to obtain enough oocytes to achieve better clinical outcomes but also to prevent the incidence of ovarian hyperstimulation syndrome (OHSS) and manage poor ovarian response, particularly in many older women (1–3). Traditionally, women’s age, serum follicle-stimulating hormone (FSH) and anti-müllerian hormone (AMH) levels, and antral follicle count (AFC) have been used for this purpose, and the starting dose of Gn in the COH cycle has been estimated (4, 5). Employing different doses of Gns for each patient is the most important clinical practice in individualized therapy (6).

The dynamic ovarian response to COH has attracted considerable attention in recent years. Different dynamic aspects of the ovarian response that correlate follicular growth to Gn have been studied, for example, ovarian sensitivity index (OSI, the dose of Gn used divided by the number of mature oocytes obtained) (7) and follicular output rate (FORT, the ratio of pre-ovulatory follicle count (14–22 mm in diameter) on human chorionic gonadotropin (hCG) day × 100/small antral follicle count (3–8 mm in diameter) at baseline (8), and the follicle-to-oocyte index (FOI, the ratio between the number of oocytes obtained and number of antral follicles at the beginning of COS) (9). The study and application of dynamic ovarian responses to COH are based on the premise that ovarian responses rely on multiple parameters. Few studies have attempted to integrate the dynamic ovarian response to different clinical parameters with the advantages of employing Gn-releasing hormone-agonist (GnRH-a) and/or GnRH-antagonists (GnRH-antag) in COH (10, 11). In this study, we investigated the correlation between OSI and multiple clinical parameters in GnRH-a and GnRH-antag cycles. The relationship between OSI and clinical outcomes of *in vitro* fertilization (IVF), especially clinical pregnancy and live birth rates, was determined.

## 2 Methods

### 2.1 Study population and design

This retrospective study analyzed the assisted reproduction files of all women in our IVF unit between January 2011 and December 2020. Data from those using the natural cycle or GnRH agonist ultra-long protocol, frozen embryo replacements, preimplantation genetic screening, or preimplantation genetic diagnosis were excluded. Only data from the first IVF treatment were included if the patients consecutively received several cycles of IVF in our unit.

### 2.2 Ethics approval

This study was approved by the Institutional Review Board (TSMH IRB/Protocol No: 18-115-B). All assisted reproductive processes were performed in accordance with the Declaration of Helsinki, Good Clinical Practice guidelines, and local regulatory requirements. All patients included in the study were treated at the IVF unit at the TUBE Fertility Clinic, Tainan, Taiwan, under a license from the Taiwan Department of Health Authority. Written consent was obtained from each patient to receive different administration modes of COH. All women receiving IVF treatments were informed about the benefits, risks, and potential adverse reactions of the entire procedure, including the different administration modes of COH (Gn, GnRH-a, and GnRH-antag). Possible risks of OHSS, allergic reactions, and local transitory effects, such as ecchymosis, itching, discomfort, and irritation were explained.

### 2.3 Controlled ovarian stimulation

The administration of GnRH-a, GnRH-antag, and Gn followed established protocols (12, 13). For the GnRH-a protocol: starting from day 3 of the preceding menstrual cycle, oral contraceptive pills (Marvelon, containing 0.03 mg ethinyl estradiol and 0.15 mg desogestrel, NV Organon, Oss, The Netherlands) were used. From day 18, a GnRH-a nasal spray (200 mg buserelin acetate, Aventis Pharma Deutschland GMBH, Frankfurt, Germany) was administered three times daily to achieve pituitary suppression. The GnRH agonist was maintained throughout the COH until the onset of hCG triggering. Gonadotropin (Gonal-f Prefilled Pen 300 IU rhFSH in 0.5 mL, Merck Serono S.p.A., Modugno, Italy) in combination with menopur (300 IU Menopur 75 IU, corresponding to 75 IU of FSH and luteinizing hormone (LH) 75 IU; Ferring GmBH, Kiel, Germany) was initiated on day 2 of the IVF cycle once pituitary suppression was achieved as manifested by serum estradiol (E2) <50 pg/mL, LH <2.5 mIU/mL, and FSH <10 mIU/mL. Intermittent injections of Gn on cycle days 2, 5, and 8 were performed in accordance with our previously established method (13). In brief, Gonal-f 300 IU in combination with 300 IU menopur was initiated on day 2 of the IVF cycle. Follow-up of ovarian follicular growth by ultrasound scanning was mostly performed on days 5 and 8–11. On days 5 and 8, if follicular growth did not meet the criteria (≥2 follicles ≥17 mm) for egg retrieval, a second and third dose of Gn injection were administered. The dosage for the second and third dose of Gn injection was based on the number and size of the follicles detected: 450 IU Gn if ≥2 follicles were >12 mm, and 600 IU Gn if most follicles were ≤12 mm.

For the GnRH-antag protocol, the third-generation GnRH antag ganirelix (orgalutron 0.25 mg, NV Organon, Oss, The Netherlands) was initiated once the ovarian follicle reached 12 mm in size on day 5 or 8 of the COH cycle. The GnRH-a was maintained throughout the COS until the day of hCG triggering. The mode of Gn administration in the GnRH-antag cycle was similar to that described for the GnRH-a cycle protocol.

Thus, two groups of patients were identified: group A received GnRH-a, and group B received GnRH-antag injections to suppress the premature LH surge. Follicular growth was detected using two-dimensional ultrasound scanning (Aloka 900, Tokyo, Japan) and performed by the same observer (C.C. Hsu) using a 5.0-MHz transvaginal transducer. The follicle diameter was calculated as the mean diameter measured in two dimensions. Serum levels of FSH, LH, progesterone (P4), and E2 on day 2 of the menstrual cycle and the day of hCG were assessed.

### 2.4 Oocyte retrieval and clinical outcomes

Oocytes were retrieved in accordance with our previously established method (13, 14). In brief, oocyte retrieval took place 36 h after triggering the final follicular maturation using 2 mg GnRH-a (Leuprolide acetate, FAMAR L’AIGLE, Saint Remy Sur Avre, France) in combination with 6,000 IU hCG (Ovidriel, Merck Serono) when two or more follicles reached ≥17 mm in diameter. Mature oocytes were fertilized *in vitro* or by intracytoplasmic sperm injection (ICSI). Fertilized pre-embryos were cultured to day 3 cleavage-stage embryos or day 5–6 blastocyst stage for embryo transfer. The number of embryos transferred was based on the age of the women: one embryo for ≤35 years old, two embryos for 35–40 years old, or three embryos for ≥40 years old. Additional embryos were cryopreserved at day 3 of cleavage stage or at the blastocyst stage. Micronized P4 (utrogesterone; Besins Healthcare, Ayutthaya, Thailand) 100 mg three times daily was used for luteal support from the day after oocyte retrieval for 15 days until pregnancy was confirmed by serum hCG determination. Clinical pregnancy was confirmed using ultrasound at 4 weeks after embryo transfer. The safety endpoints included the proportion of women with moderate/severe-grade OHSS and preventive interventions for early OHSS (i.e., cycle cancellation due to excessive ovarian response). Adverse events, such as pain or skin reactions, were also recorded during Gn and GnRH-a/GnRH-antag injections.

### 2.5 Study outcome measures

The primary outcome was the correlation between the OSI and clinical parameters, including clinical pregnancy and live birth rates during fresh embryo transfer cycles. The secondary outcomes included mature oocytes retrieved and the incidence of premature ovulation.

### 2.6 Measurement of serum hormone levels

The Beckman Coulter ACCESS immunoassay system was used in the hormone assay (UniCelDxl 800, Beckman Coulter, Brea, CA, RRID: FSH: AB_2750983, LH: AB_2750984, AMH: AB_2892998, estradiol:AB_2892997, progesterone:AB_2756883). However, FSH and LH were measured using a sequential two-step immunoenzymatic “sandwich” assay. The lowest detectable level was 0.2 IU/L, and the assay exhibited a total imprecision of ≤10% for both FSH and LH. AMH levels were measured in serum samples using a simultaneous 1-step immunoenzymatic “sandwich” assay. The assay has a limit of detection at ≤0.02 ng/mL, with total imprecision ≤10.0% at concentrations of ≥0.16 ng/mL. A competitive binding immunoenzymatic assay was used to measure serum E2 and P4 levels. The lowest detectable level of E2 was 20 pg/mL, and that of P4 was 0.10 ng/mL.

### 2.7 Statistical analysis

Continuous variables were described as mean ± standard deviation (SD), and comparisons between groups of women were conducted using the Student’s t-test. Categorical variables were expressed as frequencies and percentages, with the chi-square test applied to analyze their distributions. A generalized linear model (GLM) was employed to assess group differences while controlling for age and to evaluate the relationships between categorical and continuous variables. Correlations between the OSI and clinical parameters were analyzed using Pearson’s correlation test. Partial correlation analysis, adjusted for age, was also performed. Additionally, receiver operation characteristic (ROC) curve analysis was conducted to distinguish clinical pregnancy and live birth outcomes between the GnRH-a and GnRH-antag groups, utilizing the pROC package in R. Statistical analyses were performed using JMP Statistics version 22.0 and various R packages in R Studio.

## 3 Results

### 3.1 Participant demographics

From the data of 3,012 cases, 1,385 were excluded: 764 due to frozen embryo replacement cycles; 534, repeated treatment cycles; and 87, other exclusion factors. In total, 1627 patient data files were analyzed, of which 1,160 patients received GnRH-antag and 467 received GnRH-a IVF cycles. The demographic patterns of the infertile women are presented in **Table 1**. The average age of the study population was 36.68 ± 4.60 years, with a body mass index (BMI) of 22.32 ± 3.46 kg/m^2^. The average serum AMH was 2.67 ± 2.88 ng/mL, with AFC of 9.01 ± 6.89. Younger age and better AMH and AFC parameters were noted in GnRH-a group (**Table 1**). The cycle day 2 serum hormones FSH, E2, and LH were higher in those received GnRH-antag (**Table 2**).

**Table 1:** Demographic characteristics of the participants.

	GnRH-antagonist N = 1160	GnRH-agonist N = 467	p value	p value^1^
age	37.33 ± 4.71	35.05 ± 3.87	<.0001	
Years infertile	4.51 ± 3.3	4.69 ± 3.31	0.441	0.001
previous IVF	1.15 ± 1.94	0.64 ± 1.02	<0.001	0.008
Primary infertility	53.79% (624/1160)	57.60% (269/467)	0.141	0.891
BMI	22.44 ± 3.47	22.03 ± 3.41	0.032	0.199
AMH	2.55 ± 2.96	2.99 ± 2.64	<0.001	0.001
AFC	8.13 ± 6.43	11.28 ± 7.46	<0.001	<0.001

Data are expressed as Mean ± Standard Deviation. The statistical significance shows the results of Student’s t-test and Chi-squared test. The p value^1^ is obtained by generalized linear model (GLM) after adjustment for age.

BMI, body mass index (kg/m^2^); AMH, anti-mullerian hormone (ng/mL); AFC, antral follicle count.

**Table 2:** Endocrinology parameters in ovarian hormones and follicle growth were expressed.

	GnRH-antagonist N = 1160	GnRH-agonist N = 467	p value	p value^1^
Total dose Gn	1962.24 ± 778.87	2054.39 ± 795.30	0.031	0.009
D2 FSH	7.70 ± 3.80	5.67 ± 3.32	<0.001	<0.001
D2 E2	31.20 ± 18.71	18.07 ± 13.54	<0.001	<0.001
hCGd E2	2043.32 ± 2815.32	2052.65 ± 1914.49	0.997	0.036
Drop E2	3.71% (43/1160)	2.78% (13/467)	0.344	0.237
D2 LH	3.16 ± 2.26	1.57 ± 1.45	<0.001	<0.001
hCGd LH	3.85 ± 5.29	1.75 ± 2.16	<0.001	<0.001
Premature LH surge	3.71% (43/1160)	2.2% (10/454)	0.002	0.981
D2 P4	0.58 ± 0.44	0.56 ± 0.44	0.399	0.173
hCGd P4	1.41 ± 2.73	1.52 ± 3.41	0.689	0.855
hCGd P4 > 2	13.19% (153/1160)	3.86% (18/466)	<0.001	<0.001
Premature ovulation	0.34% (4/1160)	0.0% (0/467)	0.204	0.994
hCGd f < 11 mm	1.80 ± 2.07	1.58 ± 2.07	0.049	<0.001
hCGd f 12-14 mm	3.09 ± 3.28	4.18 ± 3.77	<0.001	0.005
hCGd f > 15 mm	5.78 ± 5.13	8.23 ± 6.18	<0.001	<0.001
hCGd Em (mm)	9.59 ± 2.72	9.83 ± 2.05	0.084	0.632
OHSS	7.84% (91/1160)	15.42% (72/467)	<0.001	0.003
Moderate to Seveve OHSS	2.76% (32/1160)	7.92% (37/467)	<0.001	0.021

Data are expressed as Mean ± Standard Deviation. The statistical significance shows the results of Student’s t-test and Chi-squared test. The p value^1^ is obtained by generalized linear model (GLM) after adjustment for age.

Gn, gonadotropin; GnRH, gonadotropin releasing hormone; D2, cycle day 2; hCGd, day of hCG injection; FSH, follicle stimulating hormone (IU/L); E2, estradiol (pg/mL); LH, luteinizing hormone (IU/L); P4, progesterone (ng/mL); f, follicle; Em, endometrium thickness; OHSS, ovarian hyperstimation syndrome.

### 3.2 Clinical response after COH using GnRH-antag or GnRH-a cycles

Elevated serum concentrations of LH, >2.5 times the baseline level and surpassing 17 IU/L, were not different between the two groups of women. Serum E2 levels on the day of hCG triggering were 2043.32 ± 2815.32 and 2052.65 ± 1914.49 pg/mL in women who received GnRH-antag and GnRH-a, respectively, with a significant difference (p = 0.036). Premature luteinization (P4 >2 ng/mL) was noted in 13.19% (153/1160) and 3.86% (18/466) of women who received GnRH-antag and GnRH-a, respectively (p <0. 001). However, the incidence of premature ovulation, indicated by the disappearance of growing follicles before oocyte retrieval, did not differ between the two groups. The number of medium-to-large-sized follicles (12–14 mm and >15 mm) and the incidence of OHSS were higher in women who received GnRH-a (**Table 2**).

### 3.3 Embryology and clinical outcomes in GnRH-antag or GnRH-a cycles

**Figure 1:**
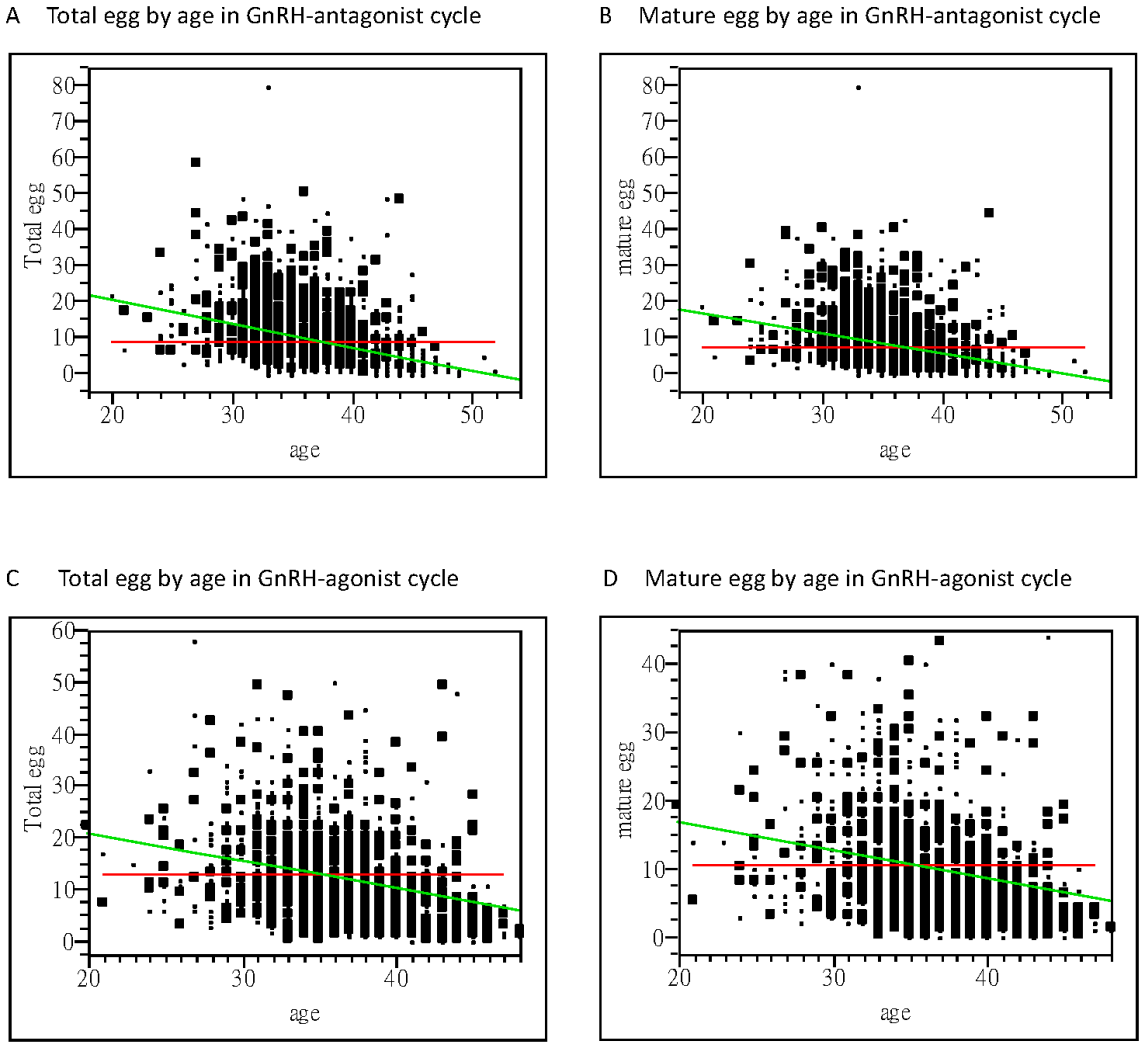
Bivariate fit of oocytes retrieved and women’s age. **(A)** A significant correlation between the total oocytes obtained and age is noted with a correlation coefficient of −0.65 using linear regression analysis (p <0.0001) in the GnRH antagonist cycle. **(B)** A significant correlation between the mature oocytes obtained and age is noted, with a correlation coefficient of −0.56 using linear regression analysis (p <0.0001) in the GnRH antagonist cycle. **(C)** A significant correlation between the total oocytes obtained and age is noted, with a correlation coefficient of−0.54 using linear regression analysis (p <0.0001) in the GnRH-agonist cycle. **(D)** A significant correlation between the mature oocytes obtained and their age was noted, with a correlation coefficient of −0.42 using linear regression analysis (p <0.0001) in the GnRH-agonist cycle. GnRH, gonadotropin-releasing hormone.

In the embryo laboratory, higher total oocyte numbers, mature oocytes, two pronuclear pre-embryos, and good embryo numbers were obtained following GnRH-a treatment cycles. Linear regression analysis of receiver operating characteristics indicated that higher numbers of oocytes were obtained from younger women, especially in the GnRH-a group (area under curve = 0.63), in comparison to area under curve = 0.52 in the GnRH-antag group. However, in both GnRH-a and GnRH-antag cycles, age was significantly correlated with total oocytes and mature oocytes (p <0.0001) (**Figure 1**).

Higher OSI of 282.67 ± 277.42 was noted in GnRH-antag treatment cycles in comparison to 201.74 ± 176.65 in GnRH-a treatment cycles (p <0.0001), indicating that higher Gns is required to stimulate one mature oocyte in GnRH-antag cycles. No difference was noted based on the FORT and FOI indices between the two groups (**Table 3**). Thus, OSI is very useful as a predictive value than FORT and FOI and was used as the ovarian response factor for further analysis with other clinical parameters.

**Table 3:** Oocytes retrieved, embryo and clinical outcomes in this study.

	GnRH-antagonist N = 1160	GnRH-agonist N = 467	p value	p value^1^
Total eggs	9.51 ± 8.04	13.32 ± 8.92	<0.001	<0.001
mature eggs	7.59 ± 6.82	10.90 ± 7.64	<0.001	<0.001
2 PN number	6.46 ± 5.52	7.91 ± 6.18	<0.001	0.016
Fertilization rate %	73.2 ± 42.5	74.7 ± 50.0	0.562	0.719
Good embryo number	4.10 ± 3.94	5.32 ± 4.61	<0.001	0.001
freeze embryo number	2.22 ± 4.20	2.48 ± 3.49	0.231	0.607
OSI	282.67 ± 277.42	201.74 ± 176.65	<0.001	<0.001
FORT	80.03 ± 89.91	79.41 ± 48.95	0.861	0.857
FOI	123.48 ± 75.20	125.73 ± 65.66	0.593	0.983
ET no.	1.84 ± 0.52	1.89 ± 0.52	0.166	0.544
Biochemical pregnancy	45.28% (470/1038)	45.45% (210/462)	0.651	0.585
Clinical pregnancy	40.85% (424/1038)	40.38% (187/462)	0.614	0.707
Live birth	32.53% (338/1038)	28.69% (136/462)	0.674	0.15

Data are expressed as Mean ± Standard Deviation. The statistical significance shows the results of Student’s t-test and Chi-squared test. The p value^1^ is obtained by generalized linear model (GLM) after adjustment for age.

PN, pronuclear; hCGd, day of hCG injection; OSI, ovarian sensitivity index, the dose of Gn used divided by number of mature oocytes obtained; FORT, follicular output rate, the ratio of pre-ovulatory follicle count (14–22 mm in diameter) on hCG day ×100/small antral follicle count (3–8 mm in diameter) at baseline.; FOI, follicle to oocyte index, the ratio between the number of oocytes obtained and the number of antral follicles at the beginning of stimulation. ET, embryo transfer.

A total of 680 women conceived following fresh embryo transfer, with a clinical pregnancy rate of 40.85% and 40.38%, and a live birth rate of 32.53% and 28.69% in the GnRH-antag and GnRH-a cycles, respectively (**Table 3**). No differences were noted in clinical pregnancy and live birth rates between the two groups.

### 3.4 The correlation between OSI and clinical parameters

**Figure 2:**
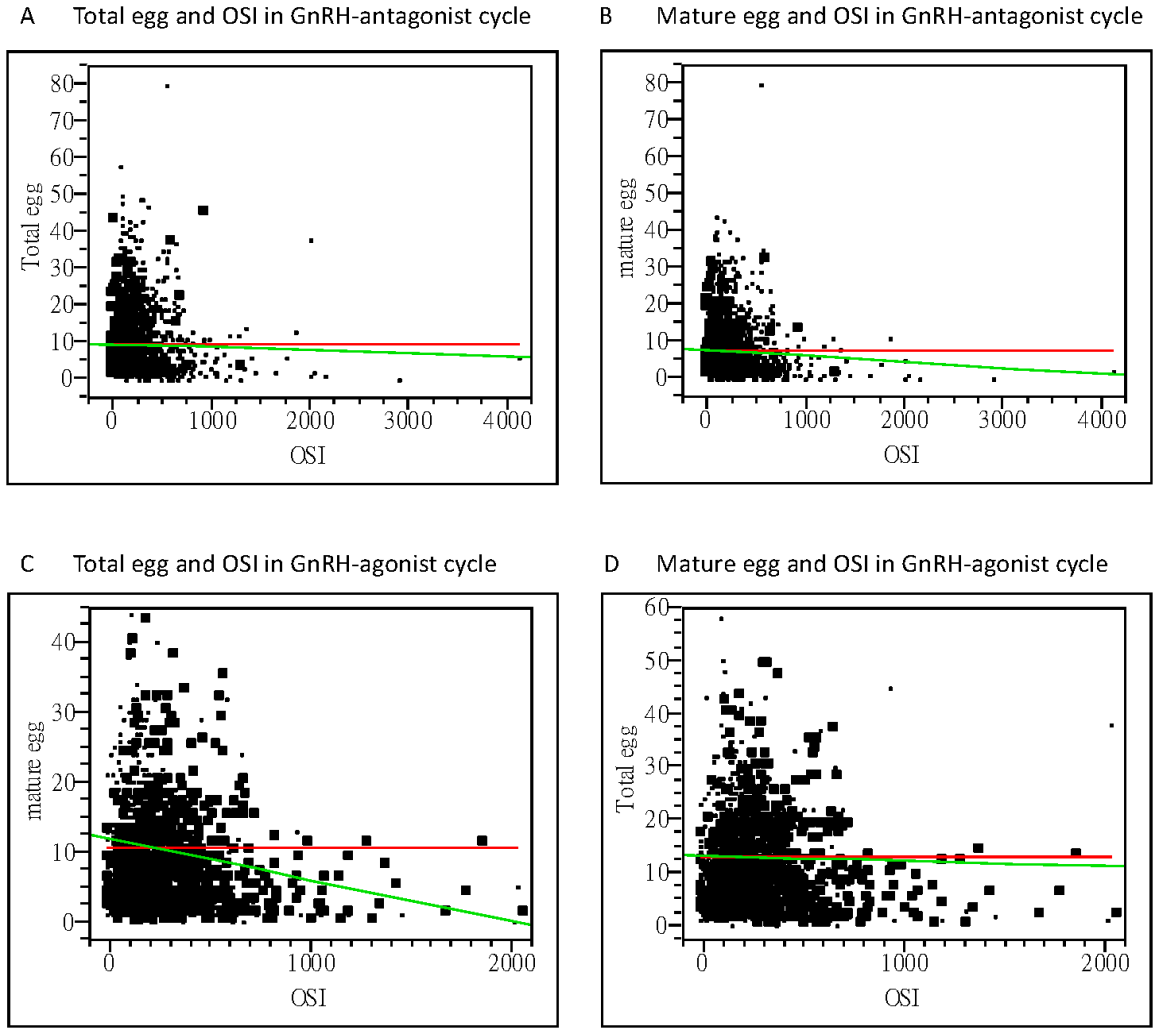
Bivariate fit of oocytes retrieved and OSI. **(A)** Correlation between the total oocytes obtained and OSI, with a correlation coefficient of −0.98 using linear regression analysis (p=0.3359) in the GnRH antagonist cycle. **(B)** Correlation between the mature oocytes obtained and OSI, with a correlation coefficient of −2.76 using linear regression analysis (p=0.0215) in the GnRH antagonist cycle. **(C)** Correlation between the total oocytes obtained and OSI, with a correlation coefficient of −0.39 using linear regression analysis (p=0.6715) in the GnRH-agonist cycle. **(D)** Correlation between the mature oocytes obtained and OSI, with a correlation coefficient of −3.13 using linear regression analysis (p=0.0034) in the GnRH-agonist cycle. OSI, ovarian sensitivity index; GnRH, gonadotropin-releasing hormone.

**Figure 3:**
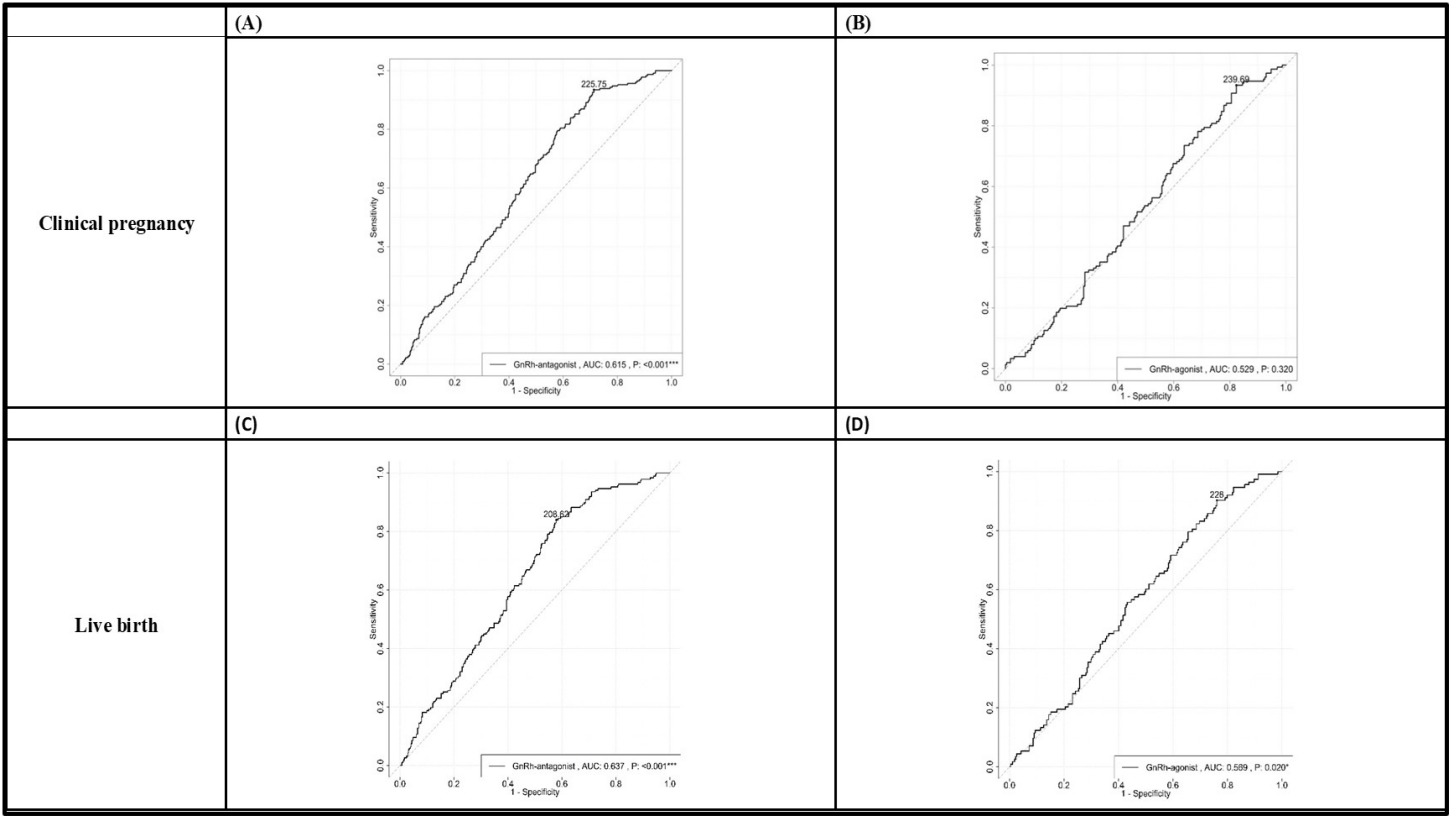
The receiver operating characteristic (ROC) curve plots demonstrated the ability to distinguish clinical pregnancy outcomes in both the GnRH antagonist group **(A)** and the GnRH agonist group **(B)**. Additionally, the plots also illustrated the ability to differentiate live birth outcomes in the GnRH antagonist group **(C)** and the GnRH agonist group **(D)**. We determined the optimal cutoff value for OSI through receiver operating characteristic (ROC) curve analysis. In the GnRH antagonist group **(A, C)**, an OSI of 225.75 significantly distinguished pregnancy from non-pregnancy (p < 0.001), with an AUC of 0.615. The sensitivity and specificity in this group were 0.935 and 0.286, respectively. It also revealed that an OSI of 208.62 significantly distinguished live births from non-live births, (p < 0.001), with an AUC of 0.637. The sensitivity and specificity in this case were 0.840 and 0.421, respectively. As for the GnRH agonist group **(B, D)**, an OSI couldn’t differentiate pregnant from non-pregnant individuals (p=0.320), while an OSI of 228 significantly distinguished live births from non-live births, (p =0.020) with an AUC of 0.569. The sensitivity and specificity were 0.903 and 0.239, respectively. GnRH, gonadotropin-releasing hormone; OSI, ovarian sensitivity index.

In all the participants, serum AMH, FSH, LH, and E2 at cycle day 2, E2, LH at hCG day, and patients’ age, fertilization rate, and signs of uncontrolled COH (including drop of E2, premature LH surge), and numbers of larger follicles were positively correlated with the OSI. Whereas BMI, serum P4 at day 2, endometrium thickness at hCG day and numbers of mature oocytes, fresh cycle embryo transfer number and dose of GnRH-antag were negatively correlated with the OSI (**Tables 4**, **5**). Among those received GnRH-antag, which represent 71.3% of our participants, the correlation between OSI and clinical parameters studied were similar to the total population. Compared with GnRH-antag cycles, higher negative correlation between numbers of mature oocytes and OSI were noted in women received GnRH-a (**Tables 4**, **5**; **Figure 2**). In the GnRH-antag group (**Figures 3A, C**), an OSI of 225.75 significantly distinguished pregnancy from non-pregnancy (p < 0.001), with an AUC of 0.615. It also revealed that an OSI of 208.62 significantly distinguished live births from non-live births, (p < 0.001), with an AUC of 0.637. As for the GnRH-a group (**Figures 3B, D**), an OSI couldn’t differentiate pregnant from non-pregnant individuals (p=0.320), while an OSI of 228 significantly distinguished live births from non-live births, (p =0.020) with an AUC of 0.569.

**Table 4:** Correlation of OSI and clinical parameters.

	Overall				GnRH-antagonist				GnRH-agonist			
	N=1627				N=1160				N=467			
	R^2^	p	R^2*^	p^*^	R^2^	p	R^2*^	p^*^	R^2^	p	R^2*^	p^*^
Age	0.060	0.022			0.037	0.219			-0.012	0.792		
BMI	-0.060	0.023	-0.063	0.012	-0.061	0.043	-0.065	0.031	-0.081	0.083	-0.080	0.085
AMH	0.060	0.027	0.087	0.002	0.103	0.001	0.125	<0.001	0.002	0.964	0.000	0.995
AFC	-0.020	0.49	0.004	0.866	0.014	0.630	0.031	0.295	0.002	0.958	0.000	0.996
Infertile years	0.010	0.64	-0.003	0.919	0.008	0.791	-0.002	0.935	0.047	0.312	0.051	0.278
Previous IVF	0.010	0.66	-0.007	0.770	-0.017	0.572	-0.031	0.311	0.059	0.208	0.062	0.186
D2 FSH	0.080	0.001	0.071	0.007	0.046	0.142	0.038	0.229	0.062	0.185	0.064	0.171
D2 E2	0.280	<0.001	0.271	<0.001	0.266	<0.001	0.265	<0.001	0.118	0.011	0.118	0.011
D2 LH	0.270	<0.001	0.269	<0.001	0.244	<0.001	0.246	<0.001	0.218	<0.001	0.218	<0.001
D2 P4	-0.090	0.001	-0.091	0.001	-0.089	0.008	-0.086	0.011	-0.127	0.007	-0.127	0.007
hCGd f <11	-0.010	0.718	-0.001	0.975	-0.025	0.411	-0.018	0.539	0.008	0.867	0.007	0.888
hCGd f 12–14	-0.030	0.311	-0.006	0.814	-0.010	0.740	0.004	0.899	0.004	0.927	0.001	0.975
hCGd f >15	0.100	<0.001	0.129	<0.001	0.156	<0.001	0.182	<0.001	0.085	0.068	0.084	0.071
hCGd E2	0.450	<0.001	0.474	<0.001	0.449	<0.001	0.471	<0.001	0.463	<0.001	0.474	<0.001
hCGd LH	0.220	<0.001	0.210	<0.001	0.219	<0.001	0.217	<0.001	0.096	0.332	0.097	0.332
hCGd P4	0.030	0.332	0.032	0.282	0.029	0.351	0.031	0.324	0.043	0.645	0.041	0.656
hCGd Em	-0.070	0.018	-0.058	0.038	-0.068	0.037	-0.062	0.054	-0.032	0.561	-0.032	0.551
Total egg	-0.020	0.343	-0.003	0.916	0.007	0.806	0.023	0.437	-0.008	0.863	-0.011	0.809
Mature egg	-0.090	0.001	-0.070	0.006	-0.040	0.183	-0.028	0.352	-0.128	0.006	-0.133	0.004
2 PN	-0.010	0.779	0.009	0.723	0.032	0.316	0.044	0.162	-0.059	0.207	-0.064	0.176
Good embryo	-0.040	0.146	-0.027	0.344	0.007	0.847	0.018	0.603	-0.082	0.090	-0.085	0.077
Total Gn dose	-0.010	0.584	-0.016	0.516	-0.027	0.373	-0.028	0.343	0.064	0.170	0.065	0.162
FORT	0.160	<0.001	0.164	<0.001	0.170	<0.001	0.170	<0.001	0.135	0.004	0.135	0.004
FOI	-0.010	0.818	-0.002	0.929	0.002	0.935	0.005	0.868	-0.038	0.409	-0.039	0.404
Freeze Embryo	0.040	0.138	0.049	0.052	0.058	0.052	0.067	0.024	-0.032	0.488	-0.033	0.474
Fresh ET No	-0.100	0.003	-0.089	0.006	-0.095	0.020	-0.092	0.024	-0.089	0.084	-0.093	0.071
Fertilization rate	0.050	0.032	0.056	0.026	0.075	0.012	0.076	0.011	0.008	0.860	0.008	0.859

The data presented consist of the coefficients and p-values for both Pearson’s correlation (coefficients: R^2^, p-values: p) and partial correlation (adjusted for age) (coefficients: R^2*^, p-values: p*) in the analysis of the relationship between OSI and the indicated clinical parameters.

BMI body mass index (kg/m^2^); AMH, anti-mullerian hormone (ng/mL); AFC, antral follicle count; Gn, gonadotropin; GnRH, gonadotropin releasing hormone; D2, cycle day 2; hCGd, day of hCG injection; FSH, follicle stimulating hormone (IU/L); E2, estradiol (pg/mL); LH, luteinizing hormone (IU/L); P4, progesterone (ng/mL); f, follicle; Em, endometrium thickness (mm); OHSS, ovarian hyperstimation syndrome; PN, pronuclear; hCGd, day of hCG injection; OSI, ovarian sensitivity index, the dose of Gn used divided by number of mature oocytes obtained; FORT, follicular output rate, the ratio of pre-ovulatory follicle count (14–22 mm in diameter) on hCG day ×100/small antral follicle count (3–8 mm in diameter) at baseline.; FOI, follicle to oocyte index, the ratio between the number of oocytes obtained and the number of antral follicles at the beginning of stimulation. ET, embryo transfer.

**Table 5:** Relationship of OSI according to the clinical parameters.

	Overall				GnRH-antagonist				GnRH-agonist			
	(N=1627)				(N=1160)				(N=467)			
	R^2^	p	R^2*^	p^*^	R^2^	p	R^2*^	p^*^	R^2^	p	R^2*^	p^*^
Primary infertility	0.057	0.035	0.079	0.08	0.073	0.054	0.095	0.075	0.014	0.713	0.036	0.805
Drop of E2	0.101	<0.001	0.102	<0.001	0.119	0.001	0.123	0.001	0.065	0.035	0.066	0.034
Premature LH surge	0.229	0.001	0.266	0.002	0.228	0.002	0.259	0.003	0.008	1	0.008	1
Premature ovulation	0.722	0.133	0.808	0.093	0.719	0.172	0.801	0.104	0.009	1	0.009	1
Elevated P4	0.063	0.066	0.064	0.065	0.07	0.335	0.071	0.314	0.017	0.184	0.017	0.199
OHSS	0.015	0.165	0.098	0.313	0.014	0.987	0.112	0.726	0.017	0.111	0.054	0.102
Biochemical pregnancy	0.011	0.015	0.044	0.051	0.013	0.029	0.071	0.077	0.008	0.324	0.014	0.375
Clinical pregnancy	0.007	0.065	0.034	0.158	0.009	0.067	0.055	0.144	0.004	0.726	0.01	0.801
Live Birth	0.009	0.029	0.047	0.092	0.012	0.042	0.07	0.106	0.006	0.35	0.023	0.429

The data are presented as the coefficients and p-values from the generalized linear model (GLM) analysis, both before (coefficients: R^2^, p-values: p) and after adjustment for age (coefficients: R^2*^, p-values: p*), to examine the relationship between OSI and the specified clinical parameters.

GnRH, gonadotropin releasing hormone; E2, estradiol (pg/mL); LH, luteinizing hormone (IU/L); P4, progesterone (ng/mL); OHSS, ovarian hyperstimation syndrome.

### 3.5 Adverse reactions

OHSS was noted in 7.84% (91/1160) and 15.42% (72/467) of patients in the GnRH-antag and GnRH-a treatment cycles, respectively. Among them, 4 and 8 patients experienced severe OHSS, 28 and 29 experienced moderate OHSS, and 59 and 35 experienced mild OHSS in those who received GnRH-antag and GnRH-a, respectively. Thus, moderate-to-severe OHSS was experienced by 2.76% (32/1160) and 7.92% (37/467) in the GnRH-antag and GnRH-a cycles, respectively. No cycle cancellation due to excessive ovarian response was noted in this study.

## 4 Discussion

In the present study, more mature oocytes and good embryos were obtained in the GnRH-a treatment cycles, which is similar to the results of most previous studies, including many systematic reviews and meta-analysis (15–19). A previous study indicated better synchronization of the follicular cohort with GnRH-a treatment and more natural recruitment of follicles in the follicular phase by employing the GnRH-antag cycle (20). They reported a strong correlation between patient age and the number of oocytes only in the GnRH-antag group (20). However, a strong correlation was noted between the woman’s age and oocytes retrieved from our patients who received either GnRH-a or GnRH-antag COH in the present study. The GnRH-antag COH has been criticized for its relatively low pregnancy rate, and it may be used as a second-line treatment (15, 21). However, our data revealed similar clinical pregnancy and live birth rates when the GnRH-a and GnRH-antag protocols were used. Under equal demographic and clinical features, previous studies have shown similar pregnancy rates with either GnRH-a or GnRH-antag protocols (20, 22). Thus, the advantage of reducing the incidence of OHSS using GnRH-antag protocols without compromising clinical outcomes is encouraged based on our results.

Previous work showed the highest correlation between ovarian response (including OSI) and AFC, AMH, LH-to-FSH ratio, age, and FSH in GnRH-antag COH cycles (10). In the present study, including GnRH-a and GnRH-antag cycles, AMH, hormone status (FSH, E2, and LH levels) on cycle day 2 before COH and E2 and LH levels on the hCG day were positively correlated with OSI. However, BMI, and number of mature oocytes were negatively correlated with OSI in these women. Our results were also different from recent reports in which OSI was inversely related to age and BMI and directly related to AMH and AFC in their GnRH-a and GnRH-antag protocols (23), and another report indicated a negative correlation between OSI and age, FSH, basal FSH/LH, and Gn total dose, and a positive correlation between OSI and AMH, AFC, total oocytes, and mature oocytes (24). However, these studies did not compare the different clinical parameters relevant to OSI separately in either the GnRH-a or GnRH-antag protocols (23, 24). Among those received GnRH-antag in the present study, the correlation between OSI and clinical parameters studied were similar to the total population as described above. Compared with GnRH-antag cycles, negative correlation between numbers of mature oocytes and OSI were noted in women received GnRH-a in our study. For the parameters of AMH and AFC in the present study, only women who received GnRH-antag showed significant correlation with OSI in AMH (correlation coefficient of 0.125; p< 0.001), with no significant correlation with OSI in AFC in either group of women. Thus, our results do not support the previous findings in which the OSI was strongly and significantly correlated with AMH and AFC (7, 25, 26).

A previous study suggested that instead of oocyte number, OSI is a better indicator of the ovarian response to Gn stimulation. For more personalized treatment, OSI has been suggested as an indicator of multiple confounding effects on oocyte number (26). The OSI has also been used as a tool to define poor, normal, and high response patterns in IVF cycles based on the long protocol GnRH-a COH (27). However, a recent study showed a marked intercycle variability of the OSI in 18% of women investigated, suggesting an intrinsic variability of ovarian sensitivity, both with the GnRH-a and GnRH-antag protocols (23). The most remarkable correlation between the OSI and clinical parameters in the present study was the demonstration of the ability to distinguish clinical pregnancy outcomes in both the GnRH-antag group and the GnRH-a group using the optimal cutoff value for OSI through receiver operating characteristic (ROC) curve analysis. Our results echo the recent study which showed a strong correlation between OSI values and the clinical pregnancy rate (23, 28). As the results were derived from data of our single institution, further investigations were warrented to confirm our finding using data from other sources. Further studies should be conducted to elucidate more consolidated clinical evidence of employing ovarian responses, including OSI, in IVF treatments, which might aid clinical decisions in the COH protocol.

There are limitations to this study, such as discrepancies in the baseline parameters of our participants; for example, the difference in the participants’ age. One factor might be the accessibility of the medicine; for example, the GnRH-a (Supremone nasal spray; Buserelin acetate, Aventis Pharma Deutschland GMBH, Frankfurt, Germany) routinely used in the long protocol for our patients who underwent IVF was no longer available in Taiwan during the last 6 years. Additionally, the mean age of women receiving IVF treatment in Taiwan has increased from 32.7 to 37.8 years between 1998 and 2021 (29). These may be important factors causing the demographic patterns of the two groups of women to differ. Moreover, retrieving ovarian follicles through vaginal puncture, especially in those suffering marked pelvic and ovarian adhesion or distorted pelvic anatomy due to huge myoma/adenomyoma, and whether or not the operating clinician retrieves oocytes from small follicles may affect OSI accuracy (10). Furthermore, low correlations between patient parameters and OSI have been related to intercycle variations in ovarian responses using the same FSH doses in the same patients (30, 31). Thus, future larger randomized controlled studies should be carried out to achieve more accuracy in the determination of ovarian response to COH, such as OSI, and towards a better elucidation of the ovarian response relevant to clinical outcomes, including clinical pregnancy and live birth rates.

In conclusion, this study reconfirmed the efficiency of both GnRH-a and GnRH-antag in suppressing premature LH surges and premature ovulation in COH for IVF treatment. Similar clinical pregnancy and live birth rates were noted when using either the GnRH-a or GnRH-antag protocols. We further demonstrated the capability of employing OSI to distinguish the clinical pregnancy and live birth outcomes in both GnRH-a and GnRH-antag cycles.

## Data Availability

The raw data supporting the conclusions of this article will be made available by the authors, without undue reservation.
